# Coexistence of Pelvic Pain, Bladder, and Bowel Symptoms in Women with Pelvic Organ Prolapse: The Effect of Transvaginal Surgery

**DOI:** 10.1007/s00192-025-06348-y

**Published:** 2025-11-08

**Authors:** Lena Schmidbauer, Bernhard Liedl, Klaus Goeschen, Aleksander Antoniewicz, Jane Kurtzman, Maren Juliane Wenk

**Affiliations:** 1 Center of Reconstructive Urogenital Surgery, Urologische Klinik, München-Planegg, Germany; 2 Formerly University of Hannover, Hannover, Germany; 3 Genitourinary Injury and Reconstructive Urology at the University of Utah, Salt Lake City, USA; 4 Department of Urology and Urological Surgery, University Medical Center Mannheim, University of Heidelberg, Theodor-Kutzer-Ufer 1-3, 68167 Mannheim, Germany

**Keywords:** Pelvic organ prolapse, Pelvic pain, Overactive bladder, Underactive bladder, Fecal incontinence, Obstructive defecation

## Abstract

**Purpose:**

To investigate the coexistence of pelvic pain, bladder, and bowel symptoms in women with pelvic organ prolapse (POP) and possible surgical symptom cure.

**Methods:**

A secondary analysis of the PROPEL study (gov-Identifier: NCT00638235) data was conducted to compare symptom prevalence preoperatively vs. 6 months after transvaginal prolapse repair with Elevate anterior and/or posterior. Symptoms were assessed with the pelvic floor distress inventory questionnaire (PFDI).

**Results:**

Two hundred seventy-seven women with symptomatic II–IV stage POP underwent mesh-supported vaginal sacrospinous ligament fixation. Of these women, 187 (67%) reported at least one pain symptom of moderate or quite a bit severity preoperatively (anterior *n* = 105, visceral *n* = 129, posterior *n* = 122). Of these women reporting pain, approximately 40–64% had coexisting symptoms of urinary urgency, daytime urinary urgency, urinary urgency-incontinence, and nocturia of moderate or quite a bit severity. A smaller part reported coexisting symptoms of underactive bladder (UAB), fecal incontinence (FI), and/or obstructive defecation (OD). Six months postoperatively, a significant reduction in the prevalence of almost all symptoms was observed. Cure rates for symptoms of overactive and underactive bladder were 65–85%, for symptoms of FI and OD 51–71%, and 58% (posterior), 85% (visceral), and 82% (anterior) for pain, respectively.

**Conclusions:**

The coexistence of bothersome pain, bladder, and bowel symptoms is common in women with POP. Therefore, POP should always be ruled out as a differential diagnosis before classifying the symptoms as interstitial cystitis/bladder pain syndrome. Women with POP and the co-existing symptoms described should be advised that surgical POP repair can resolve these symptoms in a high percentage.

**Supplementary Information:**

The online version contains supplementary material available at 10.1007/s00192-025-06348-y.

## Introduction

Pelvic organ prolapse (POP) is a common condition among women, affecting up to 50% of the population depending on its definition [1]. It is estimated that up to 13% of women will have undergone surgery for POP by the age of 80 [2]. Women with a history of pregnancies, vaginal delivery, older age, and elevated BMIs are particularly at risk [3]. POP often is co-incident with bothersome symptoms such as pelvic pain, bladder, and bowel symptoms. Specifically, common complaints are abnormal emptying of the bladder, urinary urgency, nocturia, fecal incontinence (FI), obstructed defecation (OD), and pelvic pain [4].

In 1993, chronic pelvic pain (CPP) was described as being part of the “posterior fornix syndrome” (PFS), a group of symptoms which also include nocturia, urinary urgency, and abnormal bladder emptying [5]. It was postulated that this syndrome is primarily caused by lax uterosacral ligaments (USL). The authors reported a significant symptom cure rate following USL-repair and apical vaginal reconstruction [5]. The definition of PFS was later expanded by Abendstein et al. in 2008 [6], who included anorectal disorders. They reported a significant cure rate of OD, FI, pelvic pain, and other posterior fornix symptoms following USL-repair. This was confirmed by Himmler et al. [7, 8].

The coexistence of POP, pelvic pain, and lower urinary tract symptoms (LUTS), including symptoms of overactive bladder (OAB) and underactive bladder (UAB), represents a significant challenge for clinicians. Patients with pelvic pain and LUTS, specifically urgency and frequency, are at risk of being misclassified as having interstitial cystitis/bladder pain syndrome (IC/BPS) and are often only recommended nonsurgical treatments.

We hypothesize, however, that the surgical repair of POP in women presenting with concurrent pelvic pain, urinary, and bowel complaints, can significantly improve bothersome symptoms. To date, only very limited high-quality studies have investigated this relationship. Therefore, the objective of this secondary analysis was to evaluate the prevalence of bladder and bowel symptoms in patients with pelvic pain and POP before and after prolapse repair using data from a prospective multi-center clinical trial.

## Materials and Methods

The PROPEL study (ClinicalTrials.gov-Identifier: NCT00638235) was a prospective, multicenter interventional trial, designed to primarily evaluate the long-term efficacy of the Elevate® (American Medical Systems© AMS, Minnetonka, MN) mesh kit systems for POP. The trial included female patients (all aged > 21 years) from 16 centers across the United States and Europe. Only patients with symptomatic and clinically significant POP (POP-Q stage ≥ 2) were included. This secondary analysis was conducted on the original study cohort; therefore, the exclusion criteria from the primary study apply [9, 10], and no additional exclusion criteria were imposed. Surgical repairs were conducted between May 2006 and February 2011. Institutional review board approval (e.g., Bavarian ethics committee Nr. 08021) was obtained at the time of the initial study design by all centers. The study was performed in accordance with the Declaration of Helsinki.

### Questionnaire

The study utilized the Pelvic Floor Dysfunction Inventory (PFDI) questionnaire to secondarily assess bothersome urinary and bowel/anorectal symptoms, as well as pelvic pain characteristics, including type, location, severity and frequency [11]. The PFDI uses a Likert scale with five outcomes (*no symptoms; yes, but no bother at all; somewhat bother; moderate bother; quite a bit of bother*) to score the degree of symptom bother. In the present secondary analysis, patients who scored symptom bother as “moderate” or “quite a bit” were considered to have a relevant complaint or to be clinically symptomatic (later called the composed outcome: “R2”). Accordingly, patients who indicated symptom bother as “no symptoms,” “yes but no bother at all,” or “somewhat bother” were considered to have irrelevant complaints or not to be clinically symptomatic (later called the composed outcome: “R1”). For the original study, questionnaires were completed preoperatively, and 6, 12, and 24 months postoperatively [9, 10]. The main goal of this secondary analysis was to evaluate whether the coexisting bladder and bowel symptoms in patients with POP and pelvic pain were cured by surgery (comparison preoperatively versus 6 months postoperatively).

Pain localization was classified according to PFDI items as anterior (items 1–2), visceral (items 3 and 6), or posterior (items 7 and 46), with the “total pain” group defined as pain in at least one of these regions (see Supplementary Table 1). These categories were not mutually exclusive, so patients with multiple pain sites were counted in all relevant subgroups.

The primary endpoint of this evaluation was the prevalence of coexisting “R2” (“moderate” or “quite a bit” of bother and therefore clinical relevance) pain, urinary, and bowel symptoms before and after surgery. Symptoms were classified into four main symptom domains: (1) OAB, (2) UAB, (3) FI, and (4) OD. In each domain, four questions were chosen (16 questions total). Supplementary Table 2 displays the PFDI questions and associated symptoms for each domain.

### Statistical Analysis

Since the study focused on the coexistence of urinary and bowel symptoms with pelvic pain in specific pelvic floor regions, it seemed appropriate to define a region-conditioned pain severity analogous to R2. Therefore, we consider pain in a pelvic floor region as relevant (relevant complaint; RC), when at least one symptom in that region was indicated as “R2” (“moderate” or “quite a bit” of bother), and as irrelevant (irrelevant complaint; IC), when all symptoms in that region were indicated as “R1”.

Descriptive statistics focused on symptom frequency and compared the prevalence of R2 regarding all domain symptoms preoperatively and 6 months after transvaginal surgery. On the basis of these prevalences, we calculated the cure rates (CR) for each of the domain symptoms considered. CR was defined as the change from R2 symptoms to R1 symptoms. Relative ratios (RR, preoperative/postoperative prevalence) were calculated.

Furthermore, inferential statistics were performed using the McNemar nonparametric tests. The prevalence of R2 for each domain symptom preoperatively and 6 months after transvaginal surgery was compared. The level of significance was set at 0.05.

## Results

### Baseline Demographics

The original cohort included 277 patients, who initially presented with POP stage ≥ 2. For this secondary analysis, 187 patients who initially presented with preoperative pain in the anterior region (*n* = 105, 37.9%), visceral region (*n* = 129, 46.6%), posterior region (*n* = 122, 44.0%), or total pelvic region (*n* = 187, 67.5%) were analyzed (see Supplementary Fig. 1).

The baseline demographics are shown in the original study publication [9, 10]. The following main demographics comparing the posterior/apical fixation (Elevate posterior, *n* = 135) [9] and the anterior/apical fixation (Elevate anterior, *n* = 142) [10] should be mentioned: mean age 62.5 vs. 64.7 years, mean body mass index 28.2 vs. 27.0, postmenopausal status 87.8% vs. 89.8%, and prior hysterectomy 57.6% vs. 43.8%, respectively.

### Coexisting Bladder and Bowel Symptoms

Prevalence rates of R2 for coexisting bladder symptoms (OAB and UAB, four PFDI questions each) were evaluated preoperatively for the four pain domains. The study also investigated whether and how prolapse surgery performed in the PROPEL study affected the prevalence of pain and bladder symptoms 6 months after surgery. The results are shown in Table 1.

**Table 1:** Coexisting urinary symptoms in women with pelvic organ prolapse and pelvic pain (anterior, visceral, posterior, and total): baseline and 6 months postoperatively

Coexisting urinary symptoms in women with pelvic organ prolapse and pelvic pain											
			Overactive bladder symptoms				Underactive bladder symptoms				
Regions of pain	Symptoms (bother outcome R2)	n	daytime urinary frequency	urgency	urgency incontinence	nocturia	difficulty to empty he bladder	feeling of not completely emptying the bladder	slow stream prolonged micturition	often interrupted stream	Proportion of patients with R2 pain complaints at baseline and 6 months postoperatively and corresponding cure rates
	**PFDI question** →		**PFDI 17**	**PFDI 18**	**PFDI 19**	**PFDI 27**	**PFDI 11**	**PFDI 12**	**PFDI 13**	**PFDI 14**	
**anterior pain**	baseline (%)	105	64.8	62.9	45.7	59.0	52.4	52.4	48.6	47.6	100.0
	6 months postop. (%)	101	12.9	14.9	12.9	18.8	8.9	7.9	16.8	16.8	17.80
	*p *value		**< 0.0001**	**< 0.0001**	**< 0.0001**	**< 0.0001**	**< 0.0001**	** < 0.0001**	**< 0.0001**	**< 0.0001**	**< 0.0001**
	**RR (preop/postop)**		**5.0**	**4.2**	**3.5**	**3.1**	**5.9**	**6.6**	**2.9**	**2.8**	**5.6**
	symptom cure rates		80.1	76.3	71.8	68.1	83.0	84.9	65.4	64.7	82.2
**visceral pain**	baseline (%)	129	58.9	56.6	41.9	55.8	45.7	45.0	45.0	41.1	100.0
	6 months postop. (%)	122	10.7	15.6	13.9	16.4	8.2	8.2	15.6	13.9	14.75
	*p* value		< 0.0001	< 0.0001	< 0.0001	< 0.0001	< 0.0001	< 0.0001	< 0.0001	< 0.0001	< 0.0001
	**RR (preop/postop)**		5.5	3.6	3.0	3.4	5.6	5.5	2.9	3.0	6.8
			81.8	72.4	66.8	70.6	82.1	81.8	65.3	66.2	85.3
**posterior pain**	baseline (%)	122	62.3	56.6	50.8	61.5	47.5	46.7	40.2	40.2	100.0
	6 months postop. (%)	114	17.5	16.7	13.2	19.3	10.5	13.2	12.3	14.0	41.23
	*p* value		< 0.0001	< 0.0001	< 0.0001	< 0.0001	< 0.0001	< 0.0001	< 0.0001	< 0.0001	< 0.0001
	**RR (preop/postop)**		3.6	3.4	3.8	3.2	4.5	3.5	3.3	2.9	2.4
	**symptom cure rates**		71.9	70.5	74.0	68.6	77.9	71.7	69.4	65.2	58.8
**total pain**	baseline (%)	187	57.2	56.1	45.5	55.1	42.8	42.8	40.1	36.4	100.0
	6 months postop. (%)	177	13.6	14.7	12.4	16.4	7.9	10.2	11.9	12.4	36.70
	*p* value		< 0.0001	< 0.0001	< 0.0001	< 0.0001	< 0.0001	< 0.0001	< 0.0001	< 0.0001	< 0.0001
	**RR (preop/postop)**		4.2	3.8	3.7	3.4	5.4	4.2	3.7	2.9	2.7
	**symptom cure rates**		76.2	73.8	72.7	70.2	81.5	76.2	70.3	65.9	63.3

The table shows the prevalence (%) of overactive bladder symptoms (daytime urinary frequency, urgency, urgency incontinence, nocturia) and underactive bladder symptoms (difficulty emptying the bladder, feeling of incomplete emptying, slow/prolonged stream, interrupted stream) in women with pelvic pain in different regions. Data are presented for baseline and 6 months after surgery, with *p* values for changes over time, relative risk (RR) ratios (preoperative vs. postoperative), and calculated symptom cure rates. The last column reports the proportion of patients with region-specific pelvic pain (R2, symptoms of moderate or quite a bit severity) at baseline and 6 months postoperatively, as well as corresponding cure rates

In accordance with Table 1, prevalence rates of R2 for coexisting bowel symptoms (FI and OD, four PFDI questions each) for the four pain domains and the changes 6 months postoperatively were evaluated. The results are shown in Table 2.

**Table 2:** Coexisting bowel symptoms in women with pelvic organ prolapse and pelvic pain (anterior, visceral, posterior, and total): baseline and 6 months postoperatively

Coexisting bowel symptoms in women with pelvic organ prolapse and pelvic pain										
			Fecal incontinence symptoms				Obstructive defecation symptoms			
Regions of pain	Symptoms (bother outcome R2)	n	loss of gas or stool after urgency or other warning sensations	loosing stool beyond control if it is well formed	loosing gas beyond control if it is loose or liquid	loosing gas from rectum beyond control	pushing the vagina for having a complete bowel movement	feeling to strain too hard for having a bowel movement	feeling to have not completely emptied the bowel at the end	part of bowel bulging outside during or after bowel movement
	**PFDI question **→		**PFDI 37**	**PFDI 38**	**PFDI 39**	**PFDI 40**	**PFDI 8**	**PFDI 9**	**PFDI 10**	**PFDI 45**
**anterior pain**	baseline (%)	105	31.4%	8.6%	23.8%	40.0%	41.0%	51.4%	43.8%	13.3%
	6 months postop. (%)	101	11.9%	4.0%	8.9%	16.8%	13.9%	18.8%	14.9%	5.0%
	*p* value		0.0003	0.1250	0.0009	< 0.0001	< 0.0001	< 0.0001	< 0.0001	0.0386
	**RR (preop/postop)**		2.6	2.2	2.7	2.4	2.9	2.700	2.900	2.700
	**symptom cure rates**		62.1%	53.5%	62.6%	58.0%	66.1%	63.4%	66.0%	62.4%
**visceral pain**	baseline (%)	129	25.6%	9.3%	21.7%	34.1%	37.2%	42.6%	37.2%	11.6%
	6 months postop. (%)	122	9.8%	4.1%	9.0%	16.4%	11.5%	15.6%	13.9%	3.3%
	*p* value		0.0008	0.1094	0.0033	0.0002	< 0.0001	< 0.0001	< 0.0001	0.0063
	**RR (preop/postop)**		2.6	2.3	2.4	2.1	3.2	2.7	2.7	3.5
	**symptom cure rates**		61.7%	55.9%	58.5%	51.9%	69.1%	63.4%	62.6%	71.6%
**posterior pain**	baseline (%)	122	27.9%	9.0%	23.8%	41.8%	37.7%	44.3%	40.2%	12.3%
	6 months postop. (%)	114	13.2%	3.5%	8.8%	17.5%	14.0%	20.2%	14.0%	4.4%
	*p* value		0.0062	0.1094	0.0001	0.0002	< 0.0001	< 0.0001	< 0.0001	0.0225
	**RR (preop/postop)**		2.1	2.6	2.7	2.4	2.7	2.2	2.9	2.8
	**symptom cure rates**		52.7%	61.1%	63.0%	58.1%	62.9%	54.4%	65.2%	64.2%
**total pain**	baseline (%)	187	25.7%	8.0%	21.4%	36.9%	34.2%	40.1%	36.4%	10.2%
	6 months postop. (%)	177	11.3%	2.8%	8.5%	16.9%	11.9%	16.9%	11.9%	3.4%
	*p* value		0.0003	0.0225	0.0001	< 0.0001	< 0.0001	< 0.0001	< 0.0001	0.0075
	**RR (preop/postop)**		2.3	2.9	2.5	2.2	2.9	2.4	3.1	3.0
	**symptom cure rates**		56.0%	65.0%	60.3%	54.2%	65.2%	57.9%	67.3%	66.7%

The table shows the prevalence (%) of fecal incontinence symptoms (loss of gas or stool after urgency, loss of well-formed stool beyond control, loss of loose or liquid stool beyond control, and loss of gas beyond control) and obstructive defecation symptoms (need to push on the vagina to complete bowel emptying, excessive straining, feeling of incomplete emptying, and bowel bulging during/after bowel movements) in women with pelvic pain in different regions. Data are presented for baseline and 6 months after surgery (R2, symptoms of moderate or quite a bit severity), with *p* values for changes over time and relative risk (RR) ratios (preoperative vs. postoperative)

The tables show that the prevalence of co-existing symptoms varied between 36.4% and 64.8% (OAB and UAB symptoms) and between 8.0% and 43.8% (OD and FI symptoms) preoperatively. In 15/16 PFDI questions, the relevant complaints (R2) were significantly improved by surgery in all four pain domains (all *p* < 0.05). The cure rates ranged from highest 84.9% (anterior pain/feeling of not completely emptying the bladder) to lowest 51.9% (visceral pain/losing gas from rectum beyond control), with high relative ratios accordingly. Highly significant improvement of the relevant symptoms was shown for all eight bladder symptoms (all *p* < 0.0001). Out of all PFDI questions, the improvement was statistically not significant only for PDFI 38 (loosing stool beyond control if it is well formed) due to small subgroups (anterior *p* = 0.125, visceral *p* = 0.109, posterior *p* = 0.109, respectively). Even though not significant, the cure rates for PFDI 38 were still high (between 53.5% and 61.1%).

When considering the four pain localizations, 64 individual evaluations were made (16 PFDI symptoms in four pain domains = 64 individual evaluations). In 61/64 evaluations, co-existing pain and individual symptoms were significantly improved by surgery (Tables 1 and 2).

## Discussion

In this study, we found that the coexistence of bothersome pain, bladder, and bowel symptoms is very common in women with POP. 67% reported at least one pain symptom of moderate or quite a bit severity preoperatively. Among the women who reported pain, approximately 40% to 64% also experienced concurrent symptoms such as urinary urgency, daytime frequency, urgency incontinence, and nocturia. A smaller proportion exhibited coexisting symptoms of UAB, FI, and/or OD. At 6 months postoperatively, a significant decline in the prevalence of almost all symptoms was noted. No clear correlation was observed between the location of pelvic pain (anterior, visceral, or posterior) and the pattern of coexisting urinary or bowel symptoms, as all subgroups showed similar baseline distributions and postoperative improvements.

In 2007, Pescatori [12] pointed out that patients usually seek treatment due to one main symptom, while other symptoms are present but may be latent or simply not reported by the patient unless explicitly asked. This so-called (tip of the) iceberg-concept was reviewed by Goeschen et al. [13]. In 198 patients seeking surgical treatment for POP induced pelvic pain, coexisting symptoms were evaluated: Of these women, 64% additionally suffered from urinary frequency, 34% from bladder emptying difficulties, 33% from stress urinary incontinence, 30% from obstructive defecation, 28% from urge incontinence and 22% from residual urine > 50 ml. Significant improvement of all symptoms after surgical POP-repair was observed [13]. Our findings align with previous work showing that POP is frequently associated with pelvic pain, which can markedly improve following mesh-supported POP repair. The pain outcomes reported in this study refer exclusively to pre-existing pain, and it should be noted that mesh implantation itself may, in a small proportion of patients without preoperative pain, lead to de novo pain. However, our earlier publication [11] showed that these symptoms resolve in a high percentage of cases over time.

The relationship between POP and pelvic pain was further investigated by Liedl et al. in a multicenter prospective trial. They were able to show that over two-third of patients with POP stage II–IV suffered from moderate to severe pelvic pain [11]. After surgical repair of the POP using a transvaginal mesh, pain symptoms significantly improved among affected women, with cure rates ranging from 45 to 87% depending on the location of preoperative pain [11].

These findings can best be explained when considering the pathways of pain transmission and pelvic pain development. Two different pathways were described—the visceral and the mechanical pathway [14]. The pathway which is important for the development of visceral and anterior pain is coming from the Frankenhäuser’s plexus in the parametrium in the middle of the pelvis, located lateral to the cervix. It consists of paired ganglia which can get stimulated by a descending uterus and vagina, and the pain transmits to the anterior and lateral abdominal wall, as well as the thighs and the inguinal region. It is known that this stimulation can lead to pain that can be compared to pain during labor [14]. The origin of the pain in the posterior region (often described as lower back pain) are overstretched suspensory ligaments and pelvic floor, which leads to stretching of the sacral plexus and is referred to as the mechanical pathway [14]. These pathways lead to Goeschen’s assumption that mechanical support and anatomical reconstruction of the supportive and suspensory structures can reduce/stop the prolapse-inducted stimulation of the Frankenhäuser’s plexus [11, 14].

As shown in previously published analyses of symptom subgroups, cure rates were all significant only in relation to the individual symptoms themselves (excluding the pain component) [7, 8, 11, 15, 16]. When including the pain component, we were able to show that out of 277 POP patients with a POP-Q stage ≥ II and a mean age of 63.2 ± 10.6 years, two thirds (67.5%) had at least one symptom of pelvic pain [11]. The topographical classification of the pain regions showed that *n* = 105 (37.9%) had relevant pain in the anterior, *n* = 129 (46.6%) in the visceral, and *n* = 122 (44%) in the posterior pelvic region. In further publications, we were able to show that symptoms of obstructive voiding/UAB [7], OAB [8, 15], and AD [16] co-occur frequently in women with POP-Q stages II–IV and can be cured by ligamentous POP repair up to 24 months postoperatively.

The question arises, why ligamentous POP-repair cures these symptoms as well. We pointed out that ligamentous laxities/defects are the main cause of POP [17, 18]. Meanwhile, there is evidence that POP and the hypermobility of the vaginal apex and vaginal walls stretch the fibers of the pelvic plexus. Even minor nerve alterations can cause neuroinflammation and lead to pain sensations and hypersensitivity via axon reflexes and efferent pathways [19–23]. This pain can also be transmitted to surrounding regions.

Just as pelvic pain can be triggered by anatomical changes, the bladder, and bowel complaints of these patients may also have the same anatomical cause, the laxity of the supporting and/or suspending connective tissue structures [4].

Looking at the bladder symptoms, a distinction can be made between symptoms associated with OAB and UAB. If the vaginal wall is lax, the stretch receptors of the bladder base can be stimulated which can lead to a prematurely activated micturition reflex resulting in urinary urgency, daytime urinary frequency, and nocturia (OAB) [24, 25].

Opening and closure of the bladder and anorectum can be explained by activation and relaxation of different muscle groups: rhabdosphincter, M. detrusor vesicae, M. pubococcygeus, levator plate, longitudinal muscle of the anus, M. puborectalis, and anal sphincter [4, 6, 18, 26, 27]. Lax or defect ligaments lead to elongation or shortening of muscle length, which according to Gordon [18, 26] results in a reduction of muscle force and, as a consequence, to obstructive micturition [26, 28], OD [6, 26, 27], and FI [6, 27]. The coexistence of symptom development, POP, and surgical cure by ligamentous POP repair can well be explained by these pathophysiologic pathways. Nonsurgical approaches, such as tampons or pessaries, can also provide mechanical support and help relieve both pain and urgency symptoms. Symptom improvement may therefore occur even without surgery, and conservative measures should be fully optimized before considering surgical intervention. This principle has been supported by recent studies using simulated operations [29].

The specific combination of pelvic pain and urgency is also found in the BPS/IC syndrome and therefore relevant for the following discussion. The definition of the BPS/IC is quite wide ranged. The “European society for the Study of interstitial cystitis” (ESSIC) consensus defines BPS/IC as *“chronic (*> *6 months) pelvic pain, pressure or discomfort perceived to be related to the urinary bladder accompanied by at least one other urinary symptom like persistent urge to void or frequency”* [30]. Urogenital prolapse is defined as *“confusable disease [which has to be] excluded or diagnosed by medical history and physical examination”* [30]. The diagnosis of IC/BPS is nine times more common in women than in men, so there must be a female factor, which can be the diagnosis of POP. Our study shows that these two symptoms—pelvic pain and urgency—can co-exist in women with POP and that both can be cured by surgery in a high percentage of cases, if patients are optimally selected. Therefore, POP-induced PFS should first be excluded when evaluating for IC/BPS, as its coexistence may mask the diagnostic accuracy of IC/BPS.

The strengths of the study are the multicenter prospective design, the use of a validated questionnaire and patient related outcome measures. Limitations are a reduced number of cases in the subgroups, but the statistics reveal highly significant results in the 6 months follow-up.

## Conclusion

POP is frequently associated with pain (up to 67%) as well as with bladder and bowel symptoms such as urinary urgency, daytime urinary frequency, urge incontinence, obstructive micturition, fecal incontinence, and obstructive defecation. These findings highlight that POP should always be considered (and ruled out) as a differential diagnosis when evaluating patients with interstitial cystitis/bladder pain syndrome or chronic pelvic pain. Furthermore, our study demonstrates that, in optimally selected patients, these pre-existing symptoms can be resolved by prolapse surgery in a high percentage of cases, which was the primary objective of this investigation.

## Supplementary Information

Below is the link to the electronic supplementary material.

Supplementary file1 (DOCX 18 KB)

Supplementary file2 (DOCX 65 KB)

## Data Availability

Data are available upon reasonable request.
