# Supplementary material for: Coexistence of Pelvic Pain, Bladder, and Bowel Symptoms in Women with Pelvic Organ Prolapse: The Effect of Transvaginal Surgery
Source: Int Urogynecol J. 2025 Nov 8;37(3):691–8. doi: 10.1007/s00192-025-06348-y (PMC13033018; doi:10.1007/s00192-025-06348-y)
Supplement: Supplementary file 1 — Supplementary file1 (DOCX 18 KB) [file 192_2025_6348_MOESM1_ESM.docx]

**Tables**

**Supplemental Table 1** Classification of pelvic pain

| pain region | PDFI |  |
| --- | --- | --- |
| anterior | 1 | pressure in the lower abdomen |
|  | 2 | pain in the lower abdomen |
| visceral | 3 | heaviness or dullness in the pelvic area |
|  | 6 | pelvic discomfort when standing or upon physical exertion |
| posterior | 7 | pain in the lower posterior area most days |
|  | 46 | abdominal or lower posterior area pain when straining for any reason |
| total |  | any pain in at least one of the regions (anterior, visceral or posterior) |

**Supplemental Table 1** Classification of pelvic pain (PDFI-questions)

**Supplemental Table 2** Coexisting Symptoms

| **Symptom domains** | **PDFI** |  |
| --- | --- | --- |
| Overactive bladder | 17 | daytime urinary frequency |
|  | 18 | urgency |
|  | 19 | urgency incontinence |
|  | 27 | nocturia |
| Underactive bladder | 11 | difficulty to empty the bladder |
|  | 12 | feeling of not completely emptying the bladder |
|  | 13 | slow stream prolonged micturition |
|  | 14 | often interrupted stream at urination |
| Fecal incontinence | 37 | experiencing loss of gas or still after urgency or other warning sensations |
|  | 38 | loosing stool beyond control is it is well formed |
|  | 39 | loosing gas beyond control is it is loose or liquid |
|  | 40 | loosing gas from rectum beyond control |
| Obstructive defecation | 8 | pushing the vagina for having a completing bowel movement |
|  | 9 | feeling to strain too hard for having a bowel movement |
|  | 10 | feeling to have not completely emptied the bowel at the end |
|  | 45 | part of bowel bulging outside during or after bowel movement |

**Supplemental Table 2** Coexisting Symptoms (PDFI-questions)
