# Supplementary figures and images for: Coexistence of Pelvic Pain, Bladder, and Bowel Symptoms in Women with Pelvic Organ Prolapse: The Effect of Transvaginal Surgery
Source: Int Urogynecol J. 2025 Nov 8;37(3):691–8. doi: 10.1007/s00192-025-06348-y (PMC13033018; doi:10.1007/s00192-025-06348-y)

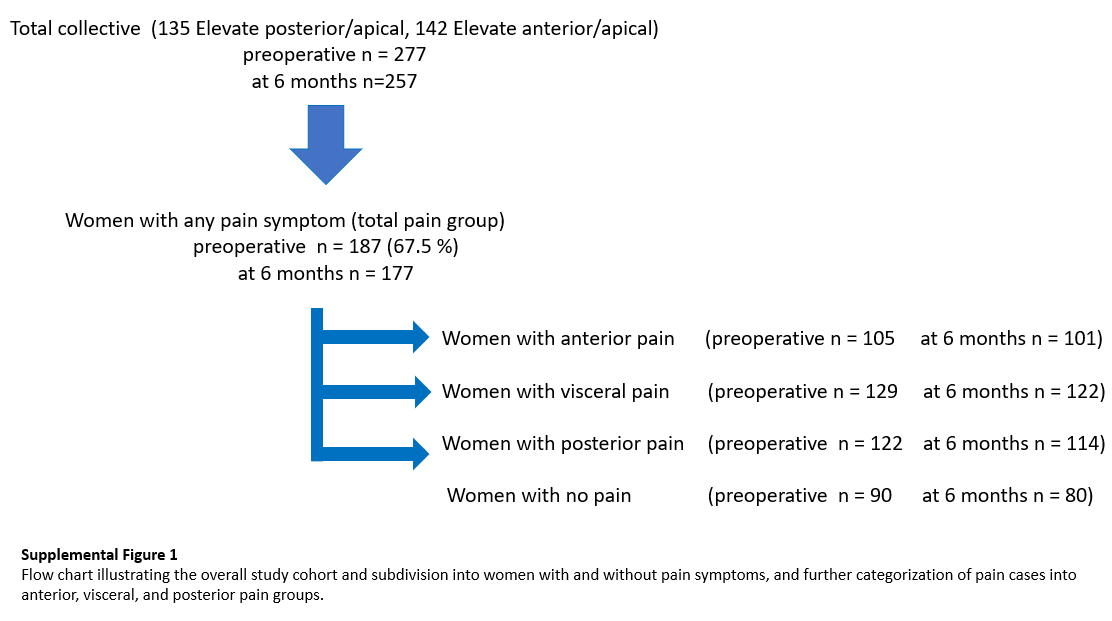

Supplement: Supplementary file 2 — Supplementary file2 (DOCX 65 KB) [file 192_2025_6348_MOESM2_ESM.docx]
